# The effect of the sphingosine‐1‐phosphate analogue FTY720 on atrioventricular nodal tissue

**DOI:** 10.1111/jcmm.12549

**Published:** 2015-04-13

**Authors:** Emmanuel E. Egom, Peter Kruzliak, Vladimir Rotrekl, Ming Lei

**Affiliations:** ^1^ Egom Clinical and Translational Research Services Halifax NS Canada; ^2^ Department of Cardiovascular Diseases International Clinical Research Center St. Anne's University Hospital and Masaryk University Brno Czech Republic; ^3^ Department of Medical Physics and Biophysics Faculty of Medicine Pavol Jozef Safarik University Kosice Slovak Republic; ^4^ Department of Biology Faculty of Medicine Masaryk University Brno Czech Republic; ^5^ Department of Pharmacology University of Oxford Oxford & Institute of Cardiovascular Sciences UK

**Keywords:** fingolimod, atrioventricular node, sphingosine‐1‐phosphate

## Abstract

The sphingosine‐1‐phosphate (S1P) receptor modulator, fingolimod (FTY720), has been used for the treatment of patients with relapsing forms of multiple sclerosis, but atrioventricular (AV) conduction block have been reported in some patients after the first dose. The underlying mechanism of this AV node conduction blockade is still not well‐understood. In this study, we hypothesize that expression of this particular arrhythmia might be related to a direct effect of FTY720 on AV node rather than a parasympathetic mimetic action. We, therefore, investigated the effect of FTY720 on AV nodal, using *in vitro* rat model preparation, under both basal as well as ischaemia/reperfusion conditions. We first look at the expression pattern of S1P receptors on the AV node using real‐time PCR. Although all three S1P receptor isoforms were expressed in AVN tissues, S1P1 receptor isoform expression level was higher than S1P2 and S1P3. The effect of 25 nM FTY720 on cycle length (CL) was subsequently studied *via* extracellular potentials recordings. FTY720 caused a mild to moderate prolongation in CL by an average 9% in AVN (*n* = 10, *P* < 0.05) preparations. We also show that FTY720 attenuated both ischaemia and reperfusion induced AVN rhythmic disturbance. To our knowledge, these remarkable findings have not been previously reported in the literature, and stress the importance for extensive monitoring period in certain cases, especially in patients taking concurrently AV node blocker agents.

## Introduction

Fingolimod (FTY720) is an orally available sphingosine‐1‐phosphate (S1P) receptor agonist, approved by the FDA for the treatment of the relapsing forms of multiple sclerosis, that induces functional change in lymphocytes and macrophages 1. In published trials, FTY720 was generally well tolerated. The most common adverse effect associated with FTY720 was a transient, dose‐dependent, usually mild negative chronotropic effect, reaching a maximum 4–5 hrs after the first dose and attenuating over time despite continued dosing and increasing blood levels 2. Similarly, FTY720 also caused dose‐dependent slowing of atrioventricular (AV) conduction with first‐and second‐degree AV block being the most common abnormality 3. Consistently, Gialafos *et al*. recently reported a female multiple sclerosis patient who developed reversible symptomatic Weckenbach type of AVB after the first two doses of FTY720 4. We hypothesize that expression of this particular arrhythmia might be related to a direct effect of FTY720 on AV node.To the best of our knowledge there are no reports demonstrating the direct effect of FTY720 on AV nodal tissue. We, therefore, investigated the effect of FTY720 on AV nodal tissue under both basal as well as ischaemia/reperfusion (I/R) conditions in isolated atrioventricular node (AVN) model.

## Materials and methods

### Dissection of the AVN

**Figure 1:**
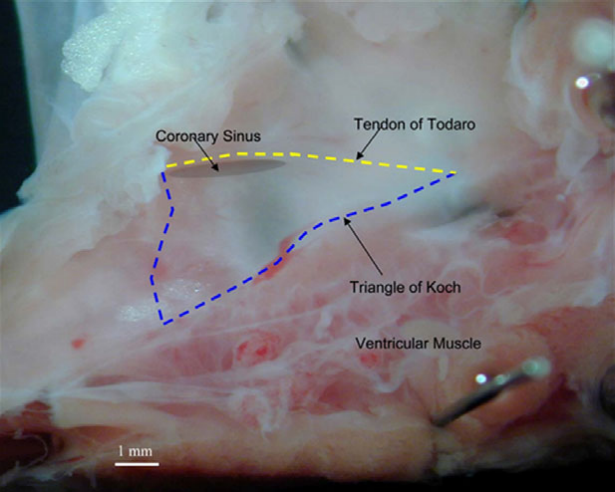
Dissection of the rat AVN tissues. The region of the AVN is outlined by a triangle; scale, 1 mm.

Whole hearts were removed and superfused with oxygenated Tyrode's solution, the Composition of which is shown in Table 1, warmed to 37°C in a water bath. The hearts were then pinned to a dissection chamber. Surrounding fatty and connective tissue were removed. A large horizontal incision was made through the ventricle to remove the apex thereby leaving only the base of the ventricles, atria and accompanying vessels. A vertical incision immediately toward the left side of the heart was made to remove the remaining left ventricle and left atrium. The aorta and pulmonary artery were removed. The inside of the right atrium was exposed by making a cut along the fold of the right atrial free wall then pinning back the free wall (endocardial surface up) onto silicon rubber. The landmark tendon of Todaro was clearly visible, which allowed two final major incisions made to remove the remaining regions of the right atrium including superior vena cava, inferior vena cava and bulk of posterior atrial wall. During the whole procedure the tissue was spontaneously beating and was continuously superfused with oxygenated Tyrode's solution, the composition of which is shown in Table 1, warmed to 37°C in a water bath. An example of a typical rat AVN preparation is shown in Figure 1.

**Table 1:** Composition of Tyrode's solution (pH 7.40) used in the SAN and AVN dissections

Chemical	Concentration (mM)	Supplier
NaCl	93	BDH
NaHCO_3_	20	BDH
Na_2_HPO_4_	1	BDH
KCl	5	BDH
CaCl_2_	2	BDH
MgSO_4_	1	Sigma‐Aldrich
Sodium acetate	20	Sigma‐Aldrich
Glucose	10	Sigma‐Aldrich
Insulin	5 units/ml	Sigma‐Aldrich

### Real‐time PCR

Real‐time PCR for S1P receptor was performed with cDNA generated from total RNA isolated from the AVN from 2 to 3 months old rats (*n* = 7). qPCR was carried out as previously described 5. Briefly, the relative abundance of selected cDNA fragments was determined with qPCR on an ABI Prism 7700 (Applied Biosystems, Warrington, Cheshire, England) and detection with a SYBR green probe. Assay primers were obtained from Qiagen (Crawley, West Sussex, UK). All runs were 40 cycles in duration. For all transcripts and samples, at least three separate measurements were made with 1 μl aliquots of each cDNA sample. The abundance of a cDNA fragment is expressed relative to the abundance of 28S (housekeeper). It was assumed that the relative abundance of a cDNA fragment reflects the relative abundance of the corresponding mRNA.

### Electrophysiology

Experiments were carried out on the AVN preparations from rats aged 2–3 months. In control groups, the AVN preparations were first perfused with modified Krebs–Henseleit Tyrode solution at rate of ~8 ml/min. for 30 min. to achieve a steady‐state condition, followed by administration of 25 nM of FTY720 (basal condition). AVN preparations were then perfused with ischaemic solution (as described below) for 20 min. and subsequently perfused with modified Krebs–Henseleit Tyrode solution (reperfusion phase) for 30 min. In the case of the FTY720 treated group, 25 nM FTY720 was added to ischaemic solution and Krebs–Henseleit buffer during reperfusion phase. The perfusion sequence was the same as for the control groups. The modified Krebs–Henseleit Tyrode solution contained (in mM): 118 NaCl, 4.7 KCl, 1.2 MgSO_4_, 1.8 CaCl_2_, 11 glucose, 25 NaHCO_3_ and 1.2 KH_2_PO4 gassed with 95% O_2_–5% CO_2_ to give a pH of 7.4. The ischaemic solution was modified based on the control perfusate buffer with the following changes: 10 mM KCl, no glucose, 1 mM NaHCO_3_, pH 6.6, and bubbled with 100% nitrogen gas for more than 30 min. before the experiment was started.

Extracellular potentials were recorded by two modified bipolar electrodes placed in the AVN preparations, as we previously described 6. Electrical signals were amplified, filtered (0.5 Hz to 1 kHz band‐pass) and digitized at a sampling frequency of 5 kHz, and stored for analysis.

### Statistical analysis

All data are reported as means ± SEM. Repeated measure one‐way anova was used to compare values of measurements obtained from the same heart before and after treatment. When anova revealed the existence of a significant difference among values, Tukey's test was applied to determine the significance of a difference between selected group means. A *P* < 0.05 was taken as an upper limit to indicate a significant difference.

## Results

**Figure 2:**
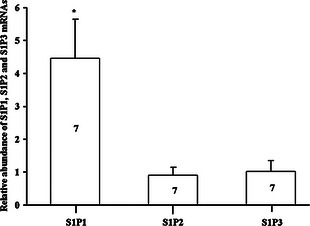
Expression profiles of S1P receptor mRNAs in rat AVN tissue. Means ± SEM shown, *P < 0.05; one‐way anova. The numbers shown in the columns are representative of the number of independent experiments.

The expression of the S1P receptor transcript pools including S1P_1_, S1P_2_ and S1P_3_ were also detected and analysed by RT‐PCR in tissues dissected from the AVN (*n* = 7 hearts). cDNA was generated from total RNA isolated from the AVN tissues. The 28S reference gene was used for data normalization. This standardized method permits comparison of relative transcript expression level. Although all three S1P receptor isoforms were expressed in AVN tissues, S1P1 receptor isoform expression level was higher than S1P2 and S1P3 in this tissue type (Fig. 2).

**Figure 3:**
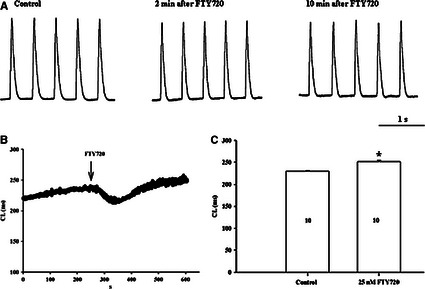
The effect of FTY720 on CL in rat AVN preparations. (**A**) Bipolar extracellular potential recording from isolated AVN preparation. (**B** and **C**) Effect of 25 nM FTY720 on CL. *P < 0.05 for the CL comparison between the control *versus* treated. The numbers shown in the columns are representative of the number of independent experiments.

The effect of 25 nM FTY720 on cycle length (CL) was studied in isolated AVN. The tissues preparations were first perfused with normal Tyrode solution at rate of ~8 ml/min. to achieve a steady‐state condition as determined by observing a stable and regular extracellular potentials *via* continual bipolar custom‐made electrode monitoring. The tissue preparations were then perfused with a Tyrode solution in which 25 nM FTY720 was added and subsequently perfused with a Tyrode solution (reperfusion phase). FTY720 (25 nM) caused a mild to moderate prolongation in CL by an average 9% in AVN (control: 230 ± 0.5 msec., FTY720: 251 ± 2 msec., *n* = 10, *P* < 0.05) preparations (Fig. 3).

**Figure 4:**
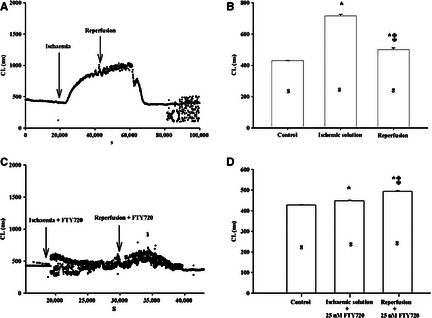
The effect of FTY720 on rhythm disturbance induced by ischaemia/reperfusion in rat AVN preparations. (**A**–**D**) Bipolar extracellular potential recording from isolated AVN preparations in the presence and absence of FTY720. *P < 0.05 for the CL comparison between the control *versus* ischaemia and reperfusion. ^‡^ *P* < 0.05 for the CL comparison between the ischaemia *versus* reperfusion. The numbers shown in the columns are representative of the number of independent experiments.

The effect of FTY720 on I/R‐induced alteration of AVN pacemaker activity was also examined in isolated AVN preparations. A bipolar custom‐made electrode was used to record ECP from central AVN and surrounding ventricular muscle. As shown in Figure 4, CL increased by 66.8% during ischaemia and by 17.7% during reperfusion (control: 429 ± 0.9 msec.; ischaemia: 716 ± 9.3 msec.; reperfusion: 501 ± 9.7, *n* = 8, *P* < 0.001). In the presence of FTY720, CL increased by 4.7% during ischaemia and by 15.4% during reperfusion (control: 427.6 ± 0.4; ischaemia: 447.5 ± 1.6; reperfusion: 493 ± 1.6, *n* = 8, *P* < 0.001). Thus, FTY720 attenuated both ischaemia‐ and reperfusion‐induced AVN rhythmic disturbance.

## Discussion

Fingolimod, a S1P receptor subtype 1, 3, 4 and 5 modulator, has been used for the treatment of patients with relapsing forms of multiple sclerosis, but atrioventricular (AV) conduction block have been reported in some patients after the first dose 7. The underlying mechanism of this AV node conduction blockade is still not well‐understood; the knowledge of which is essential for a better understanding of the clinical relevance of this side effect of FTY720. In this study, we directly look at the effect of FTY720 on AV node, using *in vito* rat model preparation, to better understand the mechanisms of the clinically observed adverse events. Our data demonstrate the important and novel finding that FTY720, an S1P analogue, directly slows down AV nodal conduction as well as effectively antagonizes conduction abnormalities induced by I/R injury, the treatment of which remains a major challenge. Our data also significantly extend earlier reports providing evidence for a role of S1P signalling in protection against I/R injury and its potential role in pre‐ and post‐conditioning 8, 9. The protective effect of ischaemic pre‐conditioning can be mimicked by exogenous administration of S1P 10. We recently found that FTY720 can prevent ischaemia‐reperfusion damage in isolated heart and sinoatrial (SA) nodes in the rat 11. We also showed that FTY720 reduces ischaemia‐ventricular arrhythmias and SA nodal dysfunction *via* activation of p21 activated kinase (Pak1), a Ser/Thr kinase downstream of small G‐proteins and Akt 10, 11, 12. How FTY720 mediates its effects is not clear. It may be related to its direct(acute) agonist effect at the S1P receptor, or potentially owing to down‐regulating the S1P receptors. Mullershausen *et al*. reported that S1P receptors activated by FTY720 retain signalling activity for hours in spite of internalization of the receptors 13. Furthermore, as Pak1/Akt phosphorylation was very rapid in our prior studies 11, it seems most likely that FTY720's effect may be related to its direct or acute effect at the S1P receptors.

Atrioventricular node is highly innervated by fibres of the left vagus and cardiac nerve, which respond to parasympathetic and sympathetic nervous system firing respectively 14. The nodal function depends among others on their balance; therefore disturbance of this equilibrium results in overactivity of the dominant system. Evidence suggests that FTY720‐induced AV block may be secondary to increased parasympathetic activity 15. The observation of a parasympathetic‐like effect of FTY720 might be because of its binding of S1P receptors located in cardiac tissue 16. This transient effect is related to a short, S1P1‐dependent activation of the G protein‐gated potassium channel IKAch in atrial myocytes, prior to internalization and/or desensitization of the S1P1 receptors by the drug 17. The direct consequence of this binding may, therefore, be a vagomimetic effect, similar to the action of acetylcholine on muscarinic receptors, causing an initial prolongation of AV impulse conduction as also observed in some patients 18, 19.

As the current experiments were carried out *in vitro* AV node preparations, our data suggest that FTY720‐induced AV node conduction block may also be a result of a direct effect of the drug on ion channels, that are expressed on the AV node. Interestingly, Yagi *et al*. have recently demonstrated that FTY720 may prolong the effective refractory period (ERP) in animal, electrically driven using a cardiac stimulator, suggesting inhibitory effects on the Na^+^ channel and/or K^+^ channel 20. Na^+^ channel blockers can increase the ERP by slowing the reactivation of fast‐Na^+^ channels, whereas K^+^ channel blockers may delay phase 3 repolarization, leading to the prolongation of the action potential duration and ERP 21. These results, therefore, suggest that FTY720 may inhibit both Na^+^ and K^+^ channels.

As hinted above, FTY720 as well as S1P may activate G protein‐coupled inwardly rectifying potassium/acetylcholine‐activated inward‐rectifying potassium (GIRK/IKACh) channel, which has been shown to be expressed in the sinoatrial and AV nodal cells and atrial muscle 22. The activation of GIRK/IKACh channel has been reported to induce AV conduction block 22, indicating that S1P‐receptor modulators may play an important role in the onset of AV conduction block.

Disturbances of cardiac rhythm, including lethal ventricular arrhythmias, are a consequence of reperfusion following pathological and/or clinical instances of myocardial ischaemia 11. This includes arrhythmias arising from re‐establishing flow after coronary spasm, cardiopulmonary bypass with ischaemic cardiac arrest, and angioplastic/thrombolytic procedures 11. The clinical occurrence and possible lethal consequences of I/R arrhythmias have prompted considerable interest in determining the mechanisms responsible and in developing therapeutic approaches for their control. Effort has thus been made to minimize the adverse arrhythmic events related to myocardial I/R injury. We have recently demonstrated that FTY720 antagonizes both bradyarrhythmias and tachyarrhythmias induced by I/R injury through Pak1/Akt signalling 11. We further showed that a disruption of Pak1 sensitizes the myocardium to I/R‐induced ventricular arrhythmias 11. We also reported that FTY720 is able to stimulate Pak1 and autophosphorylation and activities in cardiomyocytes, suggesting that FTY720 may be used for the treatment of I/R injury‐induced ventricular arrhythmias. How FTY720 mediates its antiarrhythmic effects is not clear. It may be related to its direct effect on ion channels or through its indirect effect at preventing ischaemia and thus promoting survival. Further studies are needed to fully elucidate these antiarrhythmic actions.

### Limitation of study

There may be some differences in S1P‐receptor subtypes in hearts and their signal‐transduction system between the rats and human, and pathogenesis of AV block in the rat model may not necessarily reflect that in human. In addition, the contribution of the parasympathetic nervous system effects cannot be ruled out at present *in vitro* experiment. Further electrophysiological *in vivo* studies may be needed for analysing its precise mechanisms, for example, using intracardiac electrophysiological method.

## Conclusion

The current experiments demonstrated that FTY720‐induced AV node conduction block may be a result of a direct effect of the drug on the intrinsic AV nodal electrophysiology, although an enhanced parasympsthetic action *in vivo* may not be ruled out. To our knowledge, this remarkable finding has not been previously reported in the literature, and stresses the importance for extensive monitoring period in certain cases, especially in patients taking co‐comittent AV node blocker agents.

## Conflicts of interest

Authors declare no conflict of interest.
